# Comparison of Two Measurement Methods for Scattering and Absorption Coefficients in Boron Carbide Nanodispersions

**DOI:** 10.3390/nano15201598

**Published:** 2025-10-21

**Authors:** Luca Mercatelli, Maria Raffaella Martina, Javier P. Vallejo, Luis Lugo, Elisa Sani

**Affiliations:** 1 National Institute of Optics (INO), National Research Council of Italy (CNR), Largo E. Fermi, 6, 50125 Firenze, Italy; luca.mercatelli@ino.cnr.it (L.M.); mariaraffaella.martina@unifi.it (M.R.M.); 2 Departamento de Deseño na Enxeñería, Escola de Enxeñería Industrial, Universidade de Vigo, 36310 Vigo, Spain; jvallejo@uvigo.gal; 3 Departamento de Física Aplicada, Universidade de Vigo, 36310 Vigo, Spain; luis.lugo@uvigo.es

**Keywords:** nanodispersion, spectral scattering coefficient, boron carbide nanofluid, extinction coefficient

## Abstract

Nanoparticles suspended in a liquid alter the properties of the base liquid, expanding its fields of application. Nanodispersions can have several applications in solar energy, including serving as liquid sunlight absorbers, acting as optical filters in optics, or functioning as heat transfer fluids in solar thermal applications. However, for a precise evaluation of their use in a specific field, their properties must be carefully assessed. In this work, we use two different methods for the determination of the optical scattering and absorption coefficients of a nanodispersion of boron carbide (B_4_C), and we compare the obtained results. Monochromatic measurements are performed at 635, 685, 730, and 830 nm, utilizing a technique that relies on the theory of optical scattering in an infinite medium. They are compared with spectrally resolved measurements of ballistic and total transmittance in the wavelength range of 400–850 nm, obtained using a spectrophotometer with an integrating sphere. The two methods are consistent and give results in good agreement. We also found that the mean radius of nanoparticles is higher than expected, confirming the non-negligible scattering.

## 1. Introduction

When considering renewable energy sources, solar power stands out as one of the most promising options [1]. However, effectively exploiting this abundant and freely available resource requires innovative technologies capable of harvesting sunlight and converting it into usable forms of energy—power, heat, or both. Among the emerging technologies in this field, the application of nanofluids in solar thermal systems has attracted increasing attention in recent years [2]. Nanofluids are colloidal suspensions of nanoparticles—typically on the nanometer scale—dispersed in a base fluid such as water or oil. The presence of these particles can significantly enhance the performance of solar thermal absorbers, particularly in advanced direct-absorption solar collector configurations, by improving the medium’s capacity to capture and retain solar heat [3,4]. Notably, modifications of the optical properties relevant to solar applications are achieved yet at very low nanoparticle concentrations, where the associated increase in viscosity is, in most cases, negligible [5,6,7].

Furthermore, due to their exceptionally high surface area–to–volume ratio, nanofluids exhibit superior heat transfer characteristics compared to conventional heat transfer fluids. Studies have shown that nanofluids have a significantly increased thermal conductivity compared to the base fluid [8], with enhancements of up to 45% [9]. Therefore, they have been widely investigated for heat transfer applications, like in cooling systems for electronics and different types of engines [10,11].

The determination of optical properties of the nanosuspension is of paramount importance in order to determine the minimum nanoparticle loading able to maximize optical performances with minimal concentration-dependent detrimental side effects (e.g., viscosity increase [12] and stability decrease [13]), and the information provided by the powder manufacturer is not always complete for this purpose. In fact, when nanoparticles are suspended in a fluid, they can likely form clusters so that the mean radius of the particles in the fluid could be larger than that expected from the radii of the original powders, resulting in different optical scattering and effective concentration in the fluid.

Boron carbide (B_4_C) is a dark gray–black covalent material that stands out for its high melting point, low density, and great hardness [14]. It can be considered as a member of the family of Ultra-high temperature ceramics (UHTCs). These emerging materials show higher electrical and thermal conductivities than oxide ceramics, owing to varying degrees of metallic bonding character, which entails an interesting combination of metal-like and ceramic-like properties [14]. These characteristics allow them to withstand extreme temperatures and heat flows, high levels of ionizing and non-ionizing radiations, and aggressive chemical environments, as well as large mechanical stresses [15]. UHTCs have also demonstrated ultra-refractory characteristics, as well as other promising optical properties for solar applications [16].

The effects of B_4_C nanoparticle dispersion on the physical properties of the base fluid have been scarcely investigated in the existing literature. Song et al. [17] simulated the flow field of B_4_C aqueous nanofluids in a U-shaped tube and obtained a good transmission fluid performance for a nanoparticle mass fraction of 0.8 wt%. Vallejo et al. [18] experimentally determined different thermophysical and electrical properties of a 2 wt% dispersion of B_4_C nanoparticles in a propylene glycol–water 20:80 wt% mixture, obtaining increases of up to 6.0% in thermal conductivity, up to 54% in dynamic viscosity, and up to 700% in electrical conductivity. This same nanofluid was later shown to worsen the convective heat transfer of the base fluid [19]. Sani et al. [20] experimentally investigated the optical extinction properties of B_4_C aqueous nanofluids, reporting a 99% sunlight extinction in a path length of only ~1 mm for a 0.1 wt% concentration.

In this work, the optical properties of a B_4_C nanosuspension in water are determined by means of two methods, under the assumption of high absorption and low scattering, and the results are compared. The first method considers monochromatic measurements based on the theory of optical scattering in an infinite diffusive medium [21]. The second method involves the generalization and development of a spectrophotometric method, previously proposed by some of the authors [22], through the use of the Mie theory instead of relying on the Rayleigh approximation. This new approach enables the accurate consideration of the experimental average particle size and can be applied to particles larger than the light wavelength, where the Rayleigh approximation is not applicable.

## 2. Experimental

**Sample preparation:** The boron carbide nanopowder used as a nanoadditive (purity > 99.5%) was supplied by PlasmaChem. The manufacturer also declares an average particle size of 60 nm, a specific surface area > 70 m^2^·g^−1^, and a density of 2.51 g·cm^−3^. The water used as base fluid was produced by a water purification system (Millipore, Milli-Q 185 Plus) with a resistivity higher than 18.2 MΩ cm. Dispersions at 0.05 wt% of B_4_C in water were designed using the two-step method. Quantities of each material were weighed on a balance (mod. CPA225, Sartorius AG, Göttingen, Germany) with an uncertainty of 0.01 mg. The dispersions were then sonicated for 20 min using an ultrasound probe (mod. Sonopuls HD 2200, Bandelin Electronic GmbH & Co. KG; Berlin, Germany) at a power of 200 W and a frequency of 20 kHz.

**Material characterization:** The morphology and surface topography of the B_4_C nanoparticles were characterized by atomic force microscopy (AFM) through a scanning probe microscopy (mod. Multimode 8, Veeco Instruments, New York, NY, USA). Initially, the B_4_C nanopowder was dispersed in water and then deposited onto mica substrates, allowing it to dry. Then, images were taken using the Peak Force Tapping mode and with a ScanAsyst-AIR cantilever (Silicon tip on nitride lever, tip radius of curvature < 10 nm, nominal spring constant = 0.4 N/m, frequency = 70 KHz).

Regarding the nanodispersion stability, the zeta potential of the 0.05 wt% B_4_C aqueous dispersion was evaluated using a dynamic light scattering (DLS) analyzer (Malvern Instruments, Zetasizer Nano ZS). Measurements were made at different temperatures in the range from 283.15 to 333.15 K, with a step of 10 K. High absolute values of zeta potential imply strong electrostatic forces between the dispersed particles. A value of ±30 mV is often considered a threshold for stability in aqueous dispersions [23,24,25,26].

**Scattering and absorption coefficients: monochromatic measurements.** The monochromatic measurements were composed of two parts: first, the extinction coefficient was determined via ballistic transmittance measurements; second, the absorption coefficient was obtained by means of multidistance measurements in an infinite medium composed of a highly diffusing solution (consisting of a reference pure diffuser and water) to which a small quantity of the nanosuspension of interest was added as absorber.

The extinction of radiation in a diffusing and absorbing material is composed, in fact, of scattering and absorption, and, in a generic substance showing both phenomena, the extinction of light after a distance r is defined by the Lambert–Beer law:

I(r) = I_0_ exp(−rμ_ext_) (1)

where I(r) is the transmitted light intensity measured after the path r, I_0_ is the incident intensity, and μ_ext_ is the extinction coefficient, defined by the sum of the absorption μ_a_ and scattering μ_s_ coefficients:

μ_ext_ = μ_a_ + μ_s_ (2)

The fraction of light scattered during the extinction phenomenon is defined by the albedo ω:

ω = μ_s_/μ_ext_ (3)

A reference to the diffusion equation is necessary to explain the experimental measurements of the absorption and scattering coefficients [27].

The fluence rate Φ in uniform and infinite diffusing media at a distance r from a point-like CW source [28] emitting a unit power is given by the following:

Φ(r) = (3μ′_s_/4πr) exp (−rμ_eff_) (4)

where μ′_s_ is the reduced scattering coefficient of the medium (μ′_s_ = μ_s_ (1 − g) with g anisotropy factor of the scattering function [28]) and μ_eff_ = (3 μ_a_μ′_s_)^1/2^ is the effective attenuation coefficient.

Hence, multidistance fluence measurements allow for calculating the effective attenuation coefficient μ_eff_ of the medium with a linear fit, as the slope of Equation (5), obtained by applying the natural logarithm to Equation (4):

ln [Φ (r)] = − μ_eff_ r + ln(3μ′_s_/4πr) (5)

However, it is necessary to uncouple the two coefficients, μ_a_ and μ′_s_, to determine their values. Our approach is based on repeating the measurement by changing only one coefficient in a controlled way, while keeping the other unaltered. The experimental steps were as follows: (1) the preparation of an aqueous dispersion with Intralipid-20% (Fresenius Kabi, Uppsala, Sweden), as reference diffusive medium with high scattering and negligible absorption; (2) the change in the absorption properties of the whole dispersion by adding very small, known, quantities of B_4_C nanofluid, whose little amount does not significantly modify the scattering property of the resulting fluid (B_4_C additions lower than 0.1% of the Intralipid dispersion volume). As a result, this method allowed us to modify the absorption properties of the dispersion, leaving the scattering behavior substantially unchanged. In this way, it is possible to uncouple the scattering and absorption coefficients and determine their values.

The absorption and scattering coefficients of diluted dispersions are connected to the respective values of the original dispersion by the relationship:

μ = ε·ρ(6)

where μ is the coefficient under investigation, ε is the respective value for the original non-diluted dispersion, and ρ is the concentration of the absorbing/scattering medium within the resulting dispersion, calculated as the ratio, in mass, of the nanodispersion to the resulting dispersion.

When a known quantity of absorber is added to the Intralipid dispersion, the expression of effective attenuation coefficient as a function of the added concentration of B_4_C dispersion ρ_B4C_ becomes [28]:

μ^2^_eff_(ρ_B4C_) = 3(μ_a0_ + ε_B4C_·ρ_B4C_) μ′_s0_ = 3μ_a0_·μ′_s0_ + 3μ′_s0_·ε_aB4C_·ρ_B4C_ (7)

where μ_a0_ and μ′_s0_ are the known absorption and reduced scattering coefficient of the initial Intralipid dispersion, and ε_aB4C_ the absorption coefficient of the original B_4_C dispersion, before dilution in Intralipid.

The relationship between μ^2^_eff_ and ρ_B4C_ is linear (Equation (7)). Therefore, the absorption coefficient ε_aB4C_ is obtained as the slope divided by μ′_s0_.

On the other hand, the extinction coefficient can be calculated by transmittance measurements. The explicit expression for the ballistic light transmitted through a cell of length D can be obtained by substituting the expressions (2) and (6) in Equation (1):

I(D) = I_0_ exp [− ρ_B4C_(ε_aB4C_ + ε_sB4C_) D] (8)

where ε_sB4C_ is the scattering coefficient of the original dispersion of B_4_C. As stated above for μ_eff_, the repetition of the measurements for increasing quantities of B_4_C in solution (increasing concentration) allows us to calculate the extinction coefficient from a linear equation [29]:

ln (I(D)/I_0_) = −ρ_B4C_(ε_aB4C_ + ε_sB4C_) D (9)

Regarding the experimental apparatus, the setup for multidistance fluence rate measurements in an infinite medium consists of chopped diode lasers (World Star Tech UH Series Laser Diode Modules, 635–685–730–830 nm) introduced separately into the medium via an optical fiber whose extremity radiates isotropically within the medium. A second identical optical fiber, mounted on a computer-controlled translator (MTS50\M-Z8, Thorlabs), receives the radiation at a set of fixed distances from the emitting fiber [22]. The signal received by a photomultiplier (International Light IL760) coupled to the fiber is acquired by a lock-in amplifier (EG&G model 5208 Two-Phase Lock-in Analyzer) once filtered by a Rockland mod. 452 dual Hi/Low filter.

The setup for transmittance measurements is instead composed of a calibrated length cell and the same chopped diode lasers (emitting at 635–685–730–830 nm). The light transmitted through the cell was measured with a photodiode (BPW34 PIN photodiode with Melles Griot Wide Bandwidth amplifier) and the lock-in amplifier.

The experimental setup for extinction coefficient measurement was thus similar to that of [22], with an acceptance angle of the detection system of 7 mrad. With this small acceptance angle, the unavoidable fraction of scattered received power was negligible, and the specific extinction coefficient was obtained with an error smaller than 1%.

**Scattering and absorption coefficients: spectrophotometric measurements.** The spectral scattering albedo was determined through spectrophotometric measurements using a double-beam spectrophotometer (Lambda 900, PerkinElmer) equipped with an integrating sphere for transmittance measurements (Pela1000 accessory, 150 mm diameter, input aperture radius R = 9.5 mm). A custom-designed sample cell was fabricated with a thickness of L = 5 mm and a surface area of 95 × 40 mm^2^ to provide a proper internal volume for the addition of B_4_C dispersions to pure water. This configuration also ensured low-noise measurements and sufficient data density, as described in detail below.

Our method is based on the measurement of transmittance for increasing concentrations of B_4_C (consecutive additions of known amounts of B_4_C original dispersion to pure water). For each concentration, the transmittance measurement was repeated at two different distances from the integrating sphere: with the cell in a ‘far’ position, i.e., at a distance (d_far_ = 160 mm), and with the cell in a ‘near’ position, i.e., in contact with the entrance of the integrating sphere (d_near_ = 0 mm). Thanks to the two measurements, “far” and “near”, it is possible to discriminate the scattered light that reaches the detector. In fact, in the “far” position, ballistic light is collected almost exclusively, while in the “near” position, both ballistic and scattered light within a certain angle are collected. Once the mean particle size is known, the scattering function can be determined and thus the quantity of light scattered in a certain solid angle around the forward direction.

**Figure 1:**
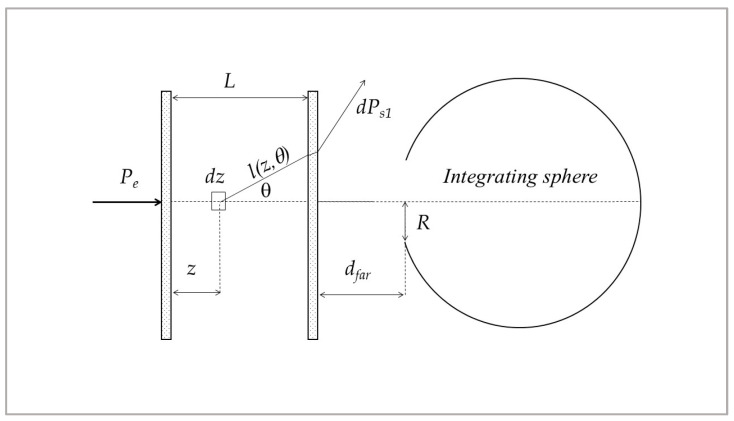
Geometry used for the model.

The expression for the scattering albedo has been obtained starting from the power P_R_ received by the integrating sphere, which is given by the following:

P_R_ = P_0_ + P_S_ (10)

where P_0_ is the ballistic component, and P_S_ is the fraction of scattered power that enters the integrating sphere. With reference to Figure 1, P_0_ is related to the input power P_e_:

P_0_ = T (θ = 0) Pe exp(−µ_ext_L) (11)

where T (θ = 0) is the transmittance of the cell windows considering the losses due to Fresnel reflections for normal incidence, while µ_ext_, defined in Equation (2) is equal to ε_ext_ρ, where ρ is the concentration of B_4_C particles (in grams per liter) and ε_ext_, their specific extinction coefficient (per millimeter per gram per liter).

The expressions which led to the determination of scattering albedo under the hypothesis of low scattering compared to absorption are derived elsewhere [22,30] and are briefly reported below.

As we have moderate values of the optical thickness τ_ext_ = μ_ext_L and low values expected for the scattering albedo, the scattered power is dominated by the contribution P_S1_ due to single scattering, thus P_S_ ≅ P_S1_.

P_S1_ is given by the following [22]:

(12) $$P_{S1}=P_{e}\int_{0}^{L}e^{-\mu_{e}z}\omega \mu_{e}\int_{0}^{\alpha }2\pi p\left(\theta \right)sin\theta T\left(\theta \right)e^{-\mu_{e}l\left(z,\theta \right)}d\theta dz=P_{0}\mu_{e}L\omega I\left(\alpha \right)$$

where p(θ) is the scattering function, l (z, θ) = (L − z)/cosθ, and

(13) $$I\left(\alpha \right)=\frac{1}{L}\int_{0}^{L}\int_{0}^{\alpha }2\pi p\left(\theta \right)sin\theta \frac{T\left(\theta \right)}{T\left(\theta =0\right)}e^{-\mu_{e}\left[\left(l-z\right)\left(\frac{1}{cos\theta }-1\right)\right]}d\theta dz$$

The angle α is the largest value of θ for which photons can enter the sphere after a single scattering event; it depends on the total reflection at the water–glass–air interface, on the distance from the sphere, and on the aperture diameter. Its values are α_near_ = 48.7° for the near position and α_far_ = 2.55° for the far one.

In the approximation that the attenuation in a path (L − z) is nearly equal to that in a path (L − z)/cosθ, which corresponds to a low scattering (small angle θ), it is possible to assume that exp(−μ_ext_(L − z)/cosθ) ≅ exp(−μ_ext_(L − z)), and then ℑ(α) becomes independent of µ_ext_, and consequently of the concentration ρ. The expression for ℑ(α) becomes the following:

(14) $$I\left(\alpha \right)≅\frac{1}{L}\int_{0}^{\alpha }2\pi p\left(\theta \right)sin\theta \frac{T\left(\theta \right)}{T\left(\theta =0\right)}dz$$

And the power received for a ρ concentration of B_4_C:

In a low scattering regime and albedo, it is ε_e_ ρ L ω ℑ(α) << 1. Therefore, we have

(15) $$P_{R}(\rho ,\alpha )≅T\left(\theta =0\right)P_{e}e^{-\varepsilon_{e}\rho L}\left[1+\varepsilon_{e}\rho L\omega I\left(\alpha \right)\right]$$

(16) $$lnP_{R}(\rho ,\alpha )≅-\varepsilon_{e}\rho L\left[1-\omega I\left(\alpha \right)\right]+ln\left[T\left(\theta =0\right)P_{e}\right]$$

From Equation (16), it is possible to obtain the measured intrinsic extinction coefficient ε_emeas_ from the slope of lnP_R_ (ρ,α) as a function of ρ (sample transmittance is measured for ten different concentrations):

(17) $$\varepsilon_{emeas}(\alpha )=\varepsilon_{e}\left[1-\omega I(\alpha )\right]$$

The expression for the albedo is as follows:

(18) $$\omega =\frac{\varepsilon_{e\;meas}\left(\alpha_{far}\right)-\varepsilon_{e\;meas}\left(\alpha_{near}\right)}{\varepsilon_{e}\left[I\left(\alpha_{near}\right)-I\left(\alpha_{far}\right)\right]}≅\frac{\varepsilon_{e\;meas}\left(\alpha_{far}\right)-\varepsilon_{e\;meas}\left(\alpha_{near}\right)}{\varepsilon_{e\;meas}(\alpha_{far})\left[I\left(\alpha_{near}\right)-I\left(\alpha_{far}\right)\right]}$$

where we assumed that ε_e_ ≅ ε_e meas_ (α_far_). To obtain ω, it is necessary to determine the values of ℑ(α). The scattering function, for a fixed wavelength, is obtained using a reverse technique: It is computed via software [22,30] once the mean radius of the particles is known from DLS measurement. Thus, the integration of Equation (14) can be performed and ℑ(α) determined, and the procedure is repeated for each wavelength. As mentioned above, this method differs from the one described previously [22,30] because now the scattering function is calculated from real particle size measurements, and we do not use the Rayleigh scattering approximation.

## 3. Results

### 3.1. AFM and DLS Measurements

**Figure 2:**
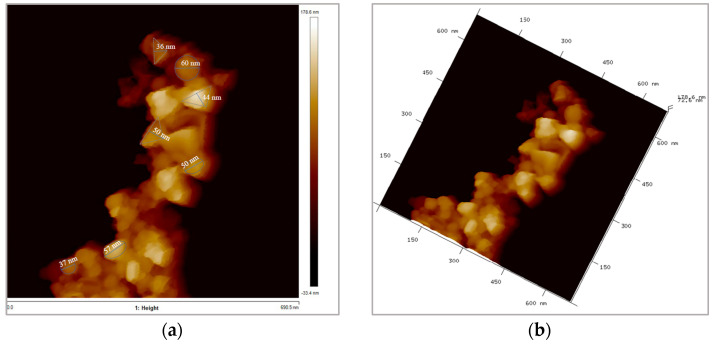
AFM topographic images of height data for B_4_C nanopowder: (**a**) 2D and (**b**) 3D.

Figure 2 shows two topographic images of height data obtained by AFM. Agglomerated but individually recognizable B_4_C nanoparticles can be observed. The majority shape is quasi-spherical, and the sizes are very similar to those reported by the manufacturer, ~60 nm. The size information provided by this analysis is also consistent with what was previously obtained from TEM and SEM analyses on the same nanoparticles [18,19,20].

**Figure 3:**
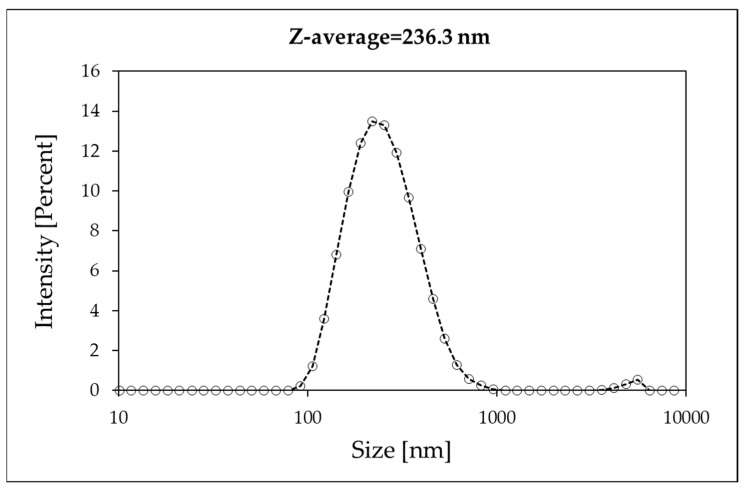
DLS measurement of the nanodispersion. The mean diameter of nanoparticles is 236.3 nm.

The DLS measurement of the nanodispersion, however, demonstrated that the mean diameter of nanoparticles is 236.3 nm (Figure 3). This is presumably a consequence of nanoparticle aggregation in the aqueous dispersion, yielding average agglomerates consisting of approximately three to four primary nanoparticles, according to the results of AFM measurements described above, which indicated that the size of an individual nanoparticle was ~60 nm. In our case, however, the bodies interacting with the optical radiation are the aggregates, and, therefore, the mean diameter determined via DLS was considered in the calculations.

**Figure 4:**
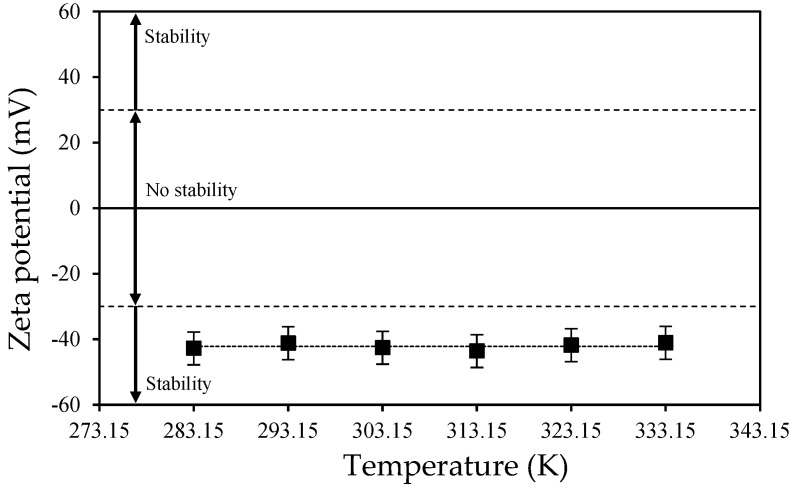
Zeta potential as a function of temperature for the 0.05 wt% B_4_C aqueous nanodispersion.

Figure 4 shows very similar zeta potential values for all the temperatures analyzed, with an average value of −42.2 mV. The values obtained are a sign of good dispersion stability over time, as per the criteria mentioned above. It is also demonstrated that this characteristic is temperature-independent within the analyzed range. These results, indicating good stability, are consistent with those shown in our previous study [20], where we reported the evolution of the Z-average size over time after preparation of B_4_C (and other) dispersions in water for two weeks. We showed there [20] that the B_4_C dispersion under static conditions maintained a similar size for several days and easily recovered its initial dispersion conditions with simple mechanical agitation.

### 3.2. Monochromatic Measurements

**Figure 5:**
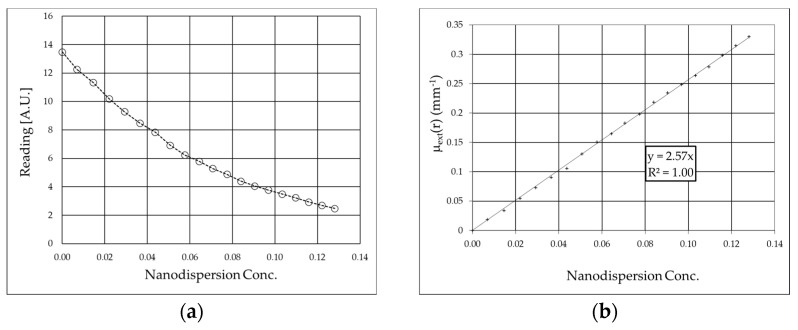
(**a**) Reading of the transmitted ballistic light (635 nm) as a function of nanodispersion concentration and (**b**) experimental values of the extinction coefficient of B_4_C at 635 nm. Concentration is defined as the mass ratio of the nanodispersion added to the overall dispersion.

The experimental values of the readings from a photodiode of ballistic monochromatic light that passes through a cell containing nanodispersion decrease as the concentration ρ of B_4_C increases, as shown in Figure 4, for the 635nm wavelength. The extinction coefficient of the original nanodispersion ε_extB4C_ is obtained by inversion of the Lambert–Beer Law (Equation (1)) as the slope of the line (Figure 5b):

ln[I_0_/I(D)]/D = ε_extB4C_ ρ (19)

where D is the cell thickness (5.13 mm).

**Figure 6:**
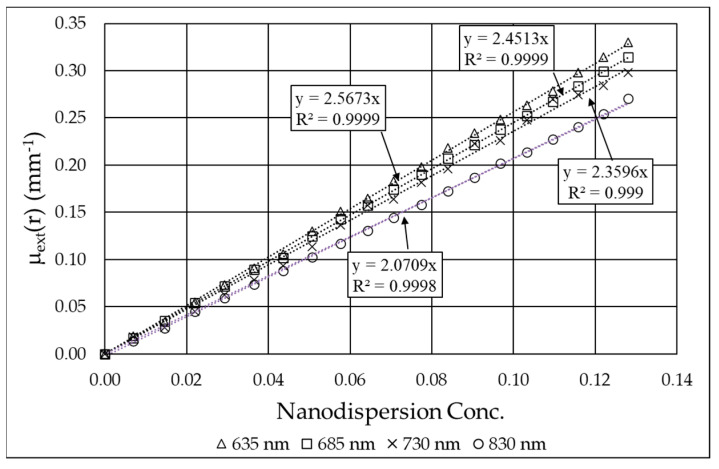
Experimental values of the extinction coefficient of B_4_C at 635, 685, 730, and 830 nm as a function of the nanodispersion concentration. Concentration is defined as the mass ratio of the nanodispersion added to the overall dispersion.

The extinction coefficient as a function of the B_4_C concentration ε_extB4C_ is 2.57 mm^−1^ at 635 nm. The values of the extinction coefficient of B_4_C calculated at 685, 730, and 830 nm are reported in Table 1, while the experimental values are reported in Figure 6.

**Figure 7:**
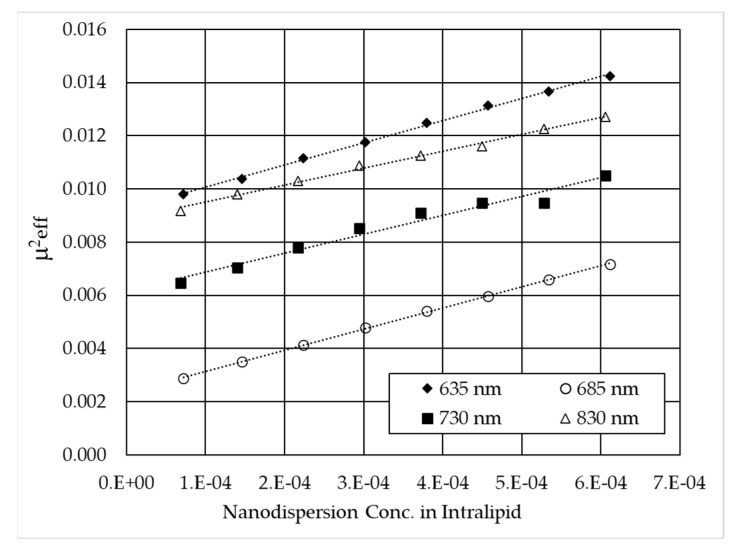
Effective attenuation coefficient μ^2^_eff_ as a function of the added concentration of B_4_C to Intralipid dispersion for the four wavelengths. The slopes represent the absorption coefficient ε_aB4C_ at a fixed wavelength. Concentration is defined as the mass ratio of the nanodispersion added to the overall dispersion.

The slope of Equation (7) represents the absorption coefficient ε_aB4C_ (once the absorption and reduced scattering coefficient of the initial Intralipid dispersion are known) and is reported in Figure 7 for the four wavelengths. With data of ε_aB4C_ and ε_extB4C_, the optical properties of the nanofluid can be fully calculated, and, in particular, the scattering albedo, defined as the ratio between the scattering and the extinction coefficient (Table 1). In Table 1, it can be observed that the absorption coefficient remains nearly constant in the examined range, changing by only 5–6%, while the scattering coefficient practically doubles from 830 nm to 635 nm. This is predictable, as the scattering of nanoparticles tends to be generally higher at shorter wavelengths unless oscillations occur due to the effect of the size parameter in Mie scattering [31,32]. On the other hand, even in the limit case of the Rayleigh approximation (i.e., particle size much smaller than wavelength), the scattering coefficient monotonically increases with decreasing wavelength.

### 3.3. Spectrophotometric Measurements

**Figure 8:**
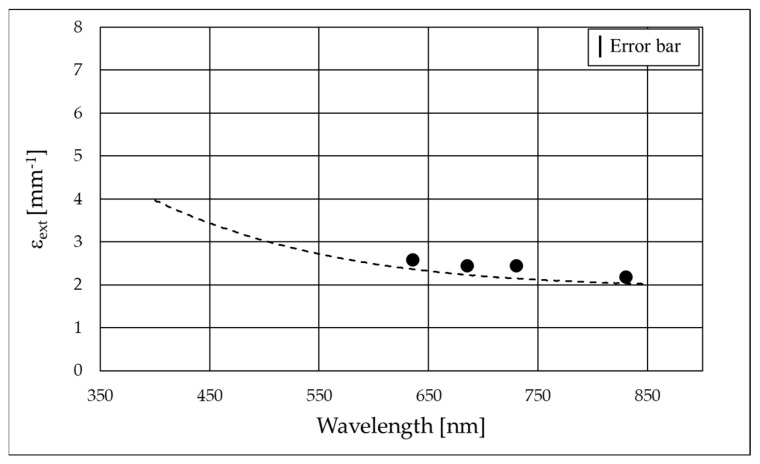
Spectral transmittance in ‘far’ position (dashed line) and monochromatic measurements of extinction coefficient (dots). The experimental uncertainties are 4% on the transmittance, which has an impact on the extinction coefficient since the errors on concentration and thickness of the cell are nearly negligible.

Spectral transmittance measurements were repeated for the two considered distances between the sample cell and the sphere entrance port. With the cell at the ‘far’ distance (d_far_ = 160 mm), only the quasi-ballistic photons (angle 2.55°) enter the sphere and are revealed, and this measurement is comparable to the monochromatic measurement of the extinction coefficient. The two measurements, spectral and monochromatic (Figure 8), show in fact a fair agreement.

**Figure 9:**
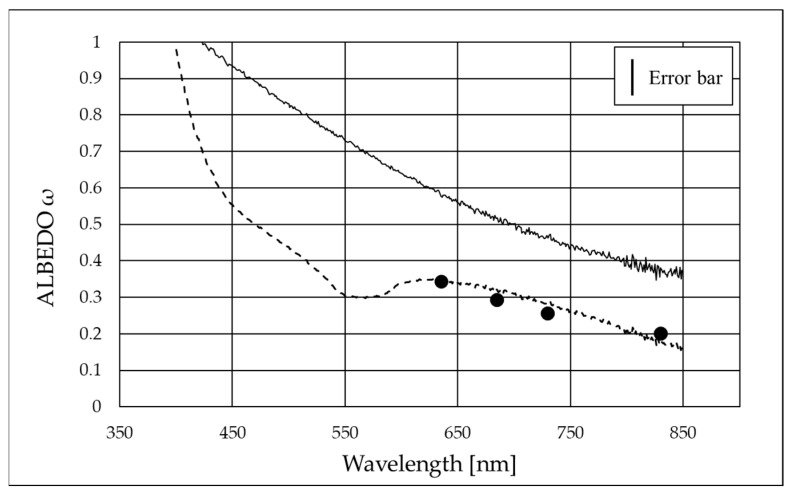
Spectral scattering albedo (dashed line) and monochromatic measurements (dots). For comparison, the albedo calculated for Rayleigh scattering (solid line) is represented.

In order to apply the procedure explained above, we calculated the spectrally resolved scattering function for a monodispersion of nanoparticles with a diameter of 236.3 nm. In fact, the DLS measurements demonstrated that although the single nanoparticle dimension was about 60 nm, the aggregates within the nanodispersion were bigger. This is a key point for applying the method correctly. In fact, if we had considered the diameter of a single particle (60 nm), we could have used the Rayleigh approximation, but this did not reflect the experimental data, as shown below. Therefore, measuring the particles as they are aggregated in the nanodispersion is of fundamental importance in order to obtain the correct scattering function in Equation (14). Then, we could calculate the angular quantities of Equation (18), obtaining the scattering albedo shown in Figure 9.

Figure 9 shows the spectral scattering albedo (dotted line) as well as the monochromatic points obtained using the monochromatic method, which demonstrate an agreement within experimental errors. This also demonstrates, for the sample under investigation, the validity of the single scattering hypothesis underlying the method. The solid line represents the spectral albedo calculated with Equation (18) in the case of Rayleigh scattering, proving that the approximation of Rayleigh scattering cannot be used in this case. This is because the size of the particles does not fulfill the Rayleigh hypothesis (i.e., it is not much smaller than the wavelength). We are therefore in the Mie scattering regime, and the Mie scattering function must be considered. In this regard, it is also worth noting that the efficiency of Mie scattering depends on the ratio between the particle size and the wavelength of the incoming radiation, described by the size parameter [31,32]. When the particle size becomes comparable to or larger than the wavelength, the efficiency shows oscillations, as evident in the dip around 550–570 nm in Figure 9, with the exact behavior being influenced by both the particle material and size.

## 4. Conclusions

A dispersion of B_4_C nanoparticles in water with a concentration of 0.05 wt% was designed for the determination of its spectral scattering and absorption coefficients. AFM analyses of the nanopowder showed a nearly spherical particle shape with a size of ~ 60 nm. The stability of the dispersion over time was confirmed by the obtained zeta potential values, which presented an average value of −42.2 mV over a wide temperature range. DLS measurements showed that the typical forms of the nanoadditive in the nanofluid are clusters consisting of 3–5 individual nanoparticles, so that the mean DLS diameter of B_4_C in the dispersion was ~240nm. This information has been proven crucial for determining the scattering behavior.

The scattering albedo was determined using two methods: a monochromatic method and a spectrophotometric method. The values obtained from both techniques showed good agreement, indicating that the simpler spectrophotometric method can be reliably applied even in cases—such as the present study—where nanoparticle-induced scattering is non-negligible, provided that the experimentally measured nanoparticle size is used to correctly compute the scattering function. Moreover, the method confirmed the validity of the single-scattering hypothesis for the nanoadditive concentrations investigated. Further studies will be conducted to evaluate the limitations of this assumption at varying nanoadditive loadings. On the other hand, a limitation of the spectrophotometric method is the need for independent knowledge of the particle diameter.

In addition, we proved that the Rayleigh approximation failed for B_4_C dispersions of this size, due to the anisotropy of the scattering function. In particular, the Rayleigh approximation overestimated the scattering albedo, giving ω = 0.35–0.75 in the range of 850–550 nm, to be compared to the values ω = 0.20–0.35 obtained from direct measurements.

Finally, a further advantage of the spectrophotometric method is the ability to obtain values of scattering and absorption coefficients in a continuous spectral range, which, in our case, went from 400 to 850 nm. This information is extremely useful for applications where the nanodispersion is subjected to spectrally continuous optical radiation, such as solar radiation.

## Figures and Tables

**Table 1:** Values of the optical coefficients of B4C calculated at selected wavelengths.

Laser Wavelength [nm]	Extinction Coefficient of B4C (ε_extB4C_) [mm^−1^]	Absorption Coefficient of B4C (ε_aB4C_) [mm^−1^]	Scattering Coefficient of B4C (ε_sB4C_) [mm^−1^]	Albedo of B4C (ω_B4C_)
635	2.57 ± 0.02	1.69 ± 0.12	0.9 ± 0.1	0.34 ± 0.06
685	2.45 ± 0.02	1.73 ± 0.12	0.7 ± 0.1	0.29 ± 0.06
730	2.36 ± 0.02	1.75 ± 0.12	0.6 ± 0.1	0.26 ± 0.06
830	2.07 ± 0.02	1.65 ± 0.12	0.4 ± 0.1	0.20 ± 0.07

## Data Availability

The data that support the findings of this study are available from the corresponding author upon reasonable request.
